# The risks of HCV infection among Brazilian crack cocaine users: incorporating diagnostic test uncertainty

**DOI:** 10.1038/s41598-018-35657-0

**Published:** 2019-01-24

**Authors:** Carolina Coutinho, Leonardo S. Bastos, Jurema Corrêa da Mota, Lidiane Toledo, Katia Costa, Neilane Bertoni, Francisco I. Bastos

**Affiliations:** 1 Institute of Scientific and Technological Communication and Information in Health (ICICT), FIOCRUZ, Rio de Janeiro, Brazil; 2 Program of Epidemiology in Public Health, Sergio Arouca National School of Public Health (ENSP), FIOCRUZ, Rio de Janeiro, Brazil; 3 Scientific Computing Program, FIOCRUZ, Rio de Janeiro, Brazil; 4 Division of Epidemiology, National Cancer Institute (INCa), Rio de Janeiro, Brazil

## Abstract

Despite the initiative by WHO and other international organizations to eliminate HCV in the medium term, hepatitis C infection is still a major public health problem. Even non-injecting drugs users who engage in harmful or addictive drug use are at greater risk of acquiring the infection, when compared to the general population. This study evaluate risk factors for HCV infection in users of crack/cocaine in Brazil, using multilevel models that incorporate variations in the sensitivity and specificity of the respective diagnostic tests. The sample included all the participants of a national survey on street crack cocaine users with serologically reactive result in the rapid test for the HCV as well as 4 non-reactive controls, matched by sex, age category, and major geographic region of residence. Multilevel logistic regression models were used, with and without incorporation of the diagnostic test’s sensitivity and specificity values. The odds of HCV infection were 85% higher among polydrug users, 7.81 times higher among injecting drug users, and 3.69 times higher in those reporting to have genital ulcers. Statistical modeling strategies that incorporate the sensitivity and specificity of diagnostic tests in challenging settings are useful for studying the association between risk factors and infection status.

## Introduction

Despite undeniable strides in the treatment of hepatitis C infection and the strategy led by the WHO (World Health Organization) to eliminate hepatitis B and C by 2030^1,2^, these infections are still a major global public health problem, especially hepatitis C, for which no vaccine exists, and for which the existing treatments are expensive, although curative. Acute HCV infection is usually asymptomatic, and an estimated 15% to 45% infected persons eliminate the virus spontaneously without any treatment. The other cases (55% to 85% of persons) develop chronic HCV infection, and among these, there is a high (15% to 30%) estimated risk of hepatic cirrhosis in the absence of therapeutic intervention^1^. According to the World Health Organization^3^, approximately 700 thousand people die per year in the world from complications of HCV infection. Due to the largely asymptomatic nature of HCV infection, many HCV-positive individuals are actually unaware of their serological status. Others have been diagnosed but are unable to obtain treatment. The lack of a quick and reliable diagnosis and the high cost and limited access to new treatments are barriers to the control and elimination of the disease^3,4^.

Parenteral infection is still the main form of HCV transmission, and an estimated 1.75 million new HCV infections occurred in the world in the year 2015 alone^5^. Persons that use illegal drugs are often exposed to increased risk of various infectious diseases, basically due to their behaviors and drug consuming habits, in addition to the risks and harms associated with the respective self-administration routes^6–9^. Various studies have reported high HCV prevalence rates, especially among people who inject drugs (PWID)^10–12^. As for non-injecting drug users (NIDU), who smoke, inhale, snort, or sniff substances like heroin, [powder] cocaine, and crack cocaine, for example, there are reports of higher HCV prevalence rates than in the general population, suggesting that there may be some other relevant form of transmission, despite persistent controversies on viral viability in straws and other self-administration paraphernalia in a context in which the risks of sexual transmission may be underestimated or imprecisely assessed^13–16^.

Epidemiological studies that assess risk factors generally use logistic regression and or other statistical modeling strategies to estimate the effect of predictors potentially associated with binary target outcomes (e.g., infection status). Less bias-prone estimates require consistent information concerning the observed outcome, in this case infection status^17^. Infection status is classically defined as the result of a diagnostic test, which contemplates specificity and sensitivity parameters in use-specific contexts^18^. Thus, the results of these analyses inadvertently incorporate the inherent validity (sensitivity and specificity) of these tests and may add bias to the resulting estimates^19^. A risk factor analysis that incorporates the diagnostic tests’ sensitivity and specificity tends to produce less skewed estimates when compared to analyses that fail to consider the respective diagnostic method’s inherent uncertainties^19^. The inherent advantages of including the specificity and sensitivity ranges of a given diagnostic test in logistic regression models have been demonstrated by some studies^17,20^.

The current study thus aims to estimate risk factors for HCV infection in users of crack and/or similar substances in Brazil, using multilevel logistic regression models that incorporate the diagnostic test’s sensitivity and specificity.

## Results

Table 1 shows the sample’s distribution by sex and age category. Of the 129 HCV-positive individuals, 98 were males (76%), and the majority for both sexes combined were in the 31 to 45-year age category (53.5%). However, the sample showed an age profile with slightly older males, in whom individuals from the most prevalent age brackets (31 to 45 years and 46 and older) concentrated 85.7% of the cases, while in women the younger age brackets were more prevalent, namely 18–30 years and 31–45 years, including 81.7% of the cases in females.

**Table 1:** Distribution of cases and controls by major geographic region, sex, and age category (matching variables). Brazil, 2012.

Geographic region	Sex	Age bracket	Cases	Controls
North	Male	18 to 30	1	4
		31 to 45	3	12
		46+	1	4
	Female	18 to 30	1	4
		31 to 45	1	4
		46+	2	8
Northeast	Male	18 to 30	3	12
		31 to 45	13	52
		46+	8	32
	Female	18 to 30	0	0
		31 to 45	6	24
		46+	0	0
Southeast	Male	18 to 30	1	4
		31 to 45	11	44
		46+	6	24
	Female	18 to 30	1	4
		31 to 45	4	16
		46+	1	6
South	Male	18 to 30	5	20
		31 to 45	18	72
		46+	8	32
	Female	18 to 30	3	12
		31 to 45	4	16
		46+	2	6
Central-West	Male	18 to 30	4	16
		31 to 45	6	24
		46+	10	40
	Female	18 to 30	2	8
		31 to 45	3	12
		46+	1	4

Table 2 shows the data on sociodemographic characteristics, substance use, risk behavior, and symptoms consistent with sexually transmitted infections, comparing cases and controls. Polydrug use was more frequent among HCV-positive individuals compared to those HCV-negative users (78.3% and 65.9%, respectively). Nearly two-thirds (62.7%) of HCV-positive individuals had used crack and/or similar drugs for more than 5 years, a statistically higher proportion than among HCV-negative individuals (52.5%). Among HCV-positive individuals, 40.3% reported having traded sex for drugs or money, compared to 27.5% in HCV-negative individuals, and the difference was statistically significant. Just over half (51.9%) of HCV-positive individuals reported having used injection drugs some time in life, a significantly higher proportion than the 13.0% in HCV-negative individuals (Table 2).

**Table 2:** Proportional distribution (%) of HCV-positive and HCV-negative crack users according to socioeconomic characteristics, drug use, risk behavior, and STI symptoms. Brazil, 2012.

Variables	Categories	Cases		Controls		p-value^*^
		n	%	n	%	
Race/color	White	27	21.3	129	25.2	0.631
	Black	25	19.7	107	20.9	
	Brown	70	55.1	262	51.3	
	Indigenous/Asian-descendant	5	3.9	13	2.5	
Schooling	Did not finish any grade	9	7.0	37	7.2	0.890
	Primary	89	69.5	362	70.6	
	Secondary	23	18.0	94	18.3	
	University	7	5.5	20	3.9	
Housing^**^	Own or family’s apartment/house	33	25.6	182	35.4	0.246
	Rented or friends’ apartment/house/room	28	21.7	98	19.1	
	Temporary housing (boarding house, hotel, shelter, etc.)	8	6.2	20	3.9	
	Other	5	3.9	14	2.7	
	Street (homeless)	55	42.6	200	38.9	
Main source of income**	Odds jobs	82	65.6	361	70.8	0.568
	Regular employment with benefits	18	14.4	55	10.8	
	Illegal activity	9	7.2	23	4.5	
	Family/partner	5	4.0	18	3.5	
	Sex worker	4	3.2	13	2.5	
	Begging	7	5.6	40	7.8	
Polydrug use	Crack /1 illegal drug	28	21.7	176	34.1	0.007
	Crack/2 or more illegal drugs	101	78.3	340	65.9	
Length of crack use	Up to 5 years	44	37.3	225	47.5	0.047
	More than 5 years	74	62.7	249	52.5	
Condom use^**^	Did not have sexual relations in the last 30 days	36	27.9	167	32.4	0.252
	Failed to use condom at least once	77	59.7	266	51.7	
	Always used condoms	16	12.4	82	15.9	
Trades sex for drugs or money	No	77	59.7	374	72.5	0.005
	Yes	52	40.3	142	27.5	
HIV result (Combination T1 + T2)	No	116	90.6	474	95.4	0.037
	Yes	12	9.4	23	4.6	
Sharing paraphernalia^**^	No	34	26.4	163	33.3	0.134
	Yes	95	73.6	327	66.7	
Lifetime use of injection drugs	No	62	48.1	449	87.0	0.000
	Yes	67	51.9	67	13.0	
Genital ulcer^**^	No	123	95.3	505	97.9	0.110
	Yes	6	4.7	11	2.1	
Genital warts**	No	124	96.1	507	98.3	0.137
	Yes	5	3.9	9	1.7	
Piercing/tattoos	No	48	37.8	226	45.1	0.138
	Yes	79	62.2	275	54.9	
Oral/gingival sores^**^	No	75	58.1	272	54.0	0.396
	Yes	54	41.9	232	46.0	

Note: Only considers valid cases, does not consider missing values.

*p-value from chi-square test.

**In the 30 days prior to the interview.

**Figure 1:**
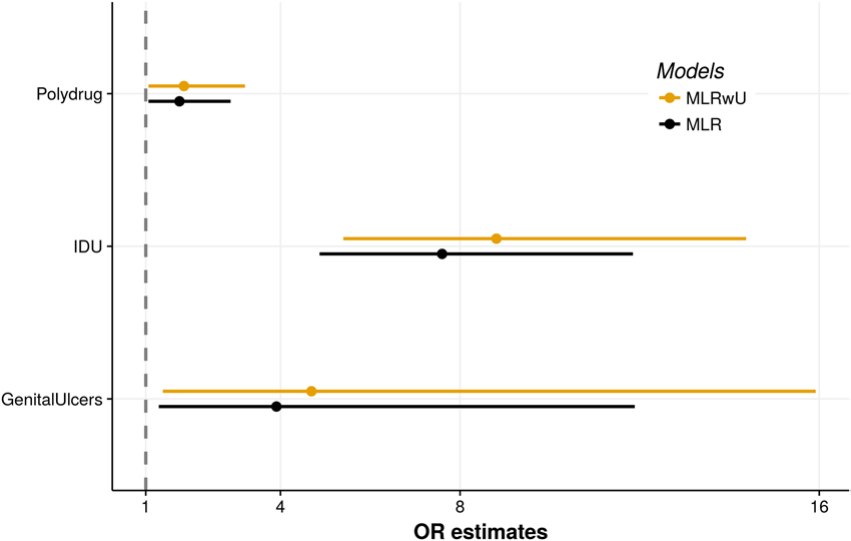
Crude and adjusted odds ratios from the final models for hepatitis C, according to the incorporation of the rapid test’s sensitivity and specificity. Brazil, 2012. MLR: Multilevel logistic regression model, MLRwU: Multilevel logistic regression model with uncertainty.

Figure 1 depicts the results of the multilevel logistic regression models, adjusted odds ratios and respective 95% confidence intervals for data without incorporation of the uncertainty potentially associated with the tests’ sensitivity and specificity. Considering the model without incorporation of uncertainty in the outcome, the odds of a positive HCV test were 75% higher in polydrug users (OR: 1.75; 95% CI:1.06–2.89), 6.60 higher in individuals that reported ever having used injection drugs (OR: 7.60; 95% CI:4.87–11.85), and 2.91 higher for individuals that reported genital ulcers (OR: 3.91; 95% CI: 1.29–11.89).

The variables in the multilevel logistic regression model that incorporated uncertainty were the same as in the traditional model. However, the odds ratios were higher for all the risk factors in the model that included uncertainty in the outcome (Fig. 1). Notice that in this model the OR can be interpreted as the individual’s likelihood of presenting positive HCV infection status. Thus, the model estimated that the odds of an individual being infected with the hepatitis C virus were 85% higher in polydrug users, compared to individuals that only used crack plus one more illegal drug (OR: 1.85; 95% CI:1.06–3.21). In relation to injection drug use, the odds of positive HCV status were 7.81 higher in persons that had used injection drugs any time in life (OR: 8.81; 95% CI:5.40–14.37). Self-reported genital ulcers were also associated with the outcome: the odds of HCV infection were 3.69 higher in individuals that reported these symptoms (OR: 4.69; 95% CI:1.38–15.92).

## Discussion

The study found that crack users are more likely to be exposed to the risk of acquiring HCV if they report lifetime injection drug use, polydrug use, and ulcerative STIs. However, corroborating the models described by Magder & Huges^17^, the model in the current study that incorporated uncertainty in the HCV test showed higher estimated odds ratios (farther from 1), evidencing that when the test’s uncertainty is not incorporated, the non-differential classification error tend to skew the estimates, thus presenting coefficients that tend to be underestimated.

The traditional multilevel logistic regression model assumes that the sensitivity and specificity in the outcome variable are 100% (or that the difference from 100% is irrelevant), that is, that there is no uncertainty in the observed outcome. This type of error may hinder the detection of associations between the explanatory variables and the outcome, since the closer to the null value the estimate is, the lower the risk attributed to it. Additionally, the confidence intervals found in the adjusted models with the incorporation of uncertainty are wider than those in the traditional model. That is, as expected, the incorporation of uncertainty is directly associated with loss of result’s precision (loss of information in favor of greater synchronization with the challenges observed in “real life situations”). This can also mean that in the traditional model, the estimated coefficient may display overestimated precision, as evidenced by narrower confidence intervals^17^. Another advantage of the multilevel logistic regression model, as it incorporates the test’s sensitivity and specificity, is the possibility of estimating the associations between the risk factors and infection status itself. In this study, since the sensitivity and specificity values adopted for the outcome (HCV test result) can be considered high [93.23% (95% CI: 89.38–96.01) and 99.07% (95% CI: 97.32–99.81), respectively], no major change was seen between the estimates generated by the traditional model and the model that incorporated uncertainty. We also added the uncertainty associated with the sensitivity and specificity values as proper elicited priors in a Bayesian multilevel logistic regression model and no major changes were observed. Results comparing all models and different inference approaches were presented in the Supplementary Material.

Lifetime injection drug use was the variable most strongly associated with HCV infection, corroborating extensive findings in the scientific literature^10–12,21^ and highlighting the relevance of HCV parenteral transmission. A prospective cohort study in three Spanish cities from 2001 to 2003 followed 305 HCV-negative persons 18 to 30 years of age who used heroin but had never injected the substance and showed that the HCV seroconversion rate was 10 times higher in persons that converted to injecting heroin than among those who never injected the drug^22^. Inciardi *et al*.^23^ conducted a qualitative study to verify changes in the pattern of cocaine use in southern Brazil and found that persons migrated from injecting cocaine to [smoked] crack use, not only because of the poor quality of the available powder cocaine (making it hard to dilute and then to inject), but also due to crack’s availability and also perhaps the influence of prevention campaigns over the years, emphasizing the risks associated with injection drug use. Thus, in Brazil today, we measure the impact of past use of injection, rather than a putative increase of rare current injection of cocaine^24^.

Some studies have found an association between length of crack use and increased risk of HCV infection^21,25^. However, in the current study, although the bivariate analysis suggested the relevance of this variable, the association did not remain in the final model. Likewise, no association was found between sharing crack-use paraphernalia and HCV infection, and no significant difference was identified in the report of this behavior when comparing HCV-positive and HCV-negative individuals. Fischer *et al*.^15^ conducted a study of 51 crack users in Toronto, Canada, with the aim of identifying HCV in the paraphernalia they used to consume the drug. The authors identified HCV in a crack pipe with a glass stem (connected to the pipe bowl) and suggested that HCV can be transmitted by sharing crack paraphernalia, and that such transmission may be modulated by the presence/absence of oral sores and the type of paraphernalia used to consume the drug. In Brazil, as shown in the National Crack Cocaine Survey^26^, most crack users recycle industrial plastic cups or bottles to smoke the drug, unlike observations in Toronto by Fischer *et al*.^15^, where most of the paraphernalia consisted of pipes and similar handmade devices. In addition, in the current study, oral and gingival sores were not associated with HCV infection, even in the bivariate analysis.

Polydrug use has been described in the literature as an important risk factor for HCV transmission, mainly in specific populations such as men who have sex with men and injecting drug users^27–30^. Polydrug use may also be associated with other risky practices such as lack of condom use during sexual relations^31,32^. A cohort study in Canada with injecting drug users found an increased risk of HCV infection among polydrug users, among individuals that used opioid in combination with cocaine/crack. The authors suggested that this increased risk of acquiring HCV results from cocaine’s stimulant effects (whether injected or smoked), which could affect decision-making processes, and which could also contribute to the increased risk of HCV transmission through shared injection paraphernalia^33^. The possible role of the combined use of opioids and stimulants is not clear, since intense, exclusive use of opioids is sometimes associated with sexual dysfunction^34^, as opposed to crack users’ intense, usually unprotected sexual activity, which may or may not be associated with the sexual transmission of HCV. Sexual transmission of HCV is still controversial^35^. In the current study, using crack and two or more other illegal drugs (other than alcohol and tobacco) was also associated with greater risk of HCV infection, suggesting that the concurrent use of different substances exposes the individual to more secondary risk factors or to risks with more complex determination (e.g., modulation of sexual behavior by different psychoactive substances or different patterns of shared use), since different substances can be administered by different routes and necessarily have specific physiological effects and associated behaviors, sometimes with synergistic interaction (as in the example of secondary metabolites from the simultaneous use of cocaine and alcohol, like cocaethylene^36^), and other times with conflicting or unpredictable effects.

In general, blood (whether contaminated or not) can penetrate the genital epithelium more readily in the presence of ulcerations or micro-lacerations. In some circumstances, these small exposures can be sufficient for HCV infection^37^. A study of 1,257 non-injecting drug users who were patients at an STI treatment clinic in Baltimore in 1987 found an HCV point prevalence of 9.7%, suggesting that the virus can be transmitted sexually in these patients^38^. Other studies^39,40^ also point to the possibility of HCV sexual transmission, especially relevant for non-injecting drug users (since parenteral exposure tends to confound any other risk factors^41,42^) with other STIs, whether ulcerative or non-ulcerative, such as herpes, gonorrhea, syphilis, and trichomoniasis. The current study also found an association between genital ulcer and HCV infection in the study population, which could correspond to a cofactor associated with unprotected sexual relations.

### Limitations

The article used data from a cross-sectional study. In other words, although it identified risk factors statistically associated with HCV infection, it is not possible to establish the directionality of such associations. The observed outcome, HCV infection, was determined by a single rapid test, and given the nature of the virus and its dynamic in the host organism (e.g., the possibility of a partial response and/or gradual establishment), another test with a different methodology is recommended to confirm the diagnosis. Thus, there may be persistent classification errors, with false-positives and/or false-negatives. However, by incorporating the uncertainty associated with the diagnostic test into the subsequent models, the findings prove more robust, reinforcing their consistency.

Since the primary objective of the Brazilian National Crack Cocaine Survey was not to identify risk factors associated with HCV, but to characterize the profile of crack users in open drug use scenes, some of the questions used time intervals that are common to other epidemiological surveys (“lifetime use”, “in the last 12 months”, and “in the 30 days prior to the interview”), and thus some variables potentially associated with HCV infection could not be assessed more comprehensively and precisely (in relation to time). Thus, the results presented here refer to crack users 18 years or older who frequently smoked crack in pipes in open scenes (that is, necessarily excluding other populations such as users that consume crack in private homes, squatter camps, the prison system, etc.) and residing in Brazilian state capitals in 2011–2012.

Other limitation to be considered here refers to the need to match cases and controls by major geographic region of the country (macro region), since the matching by state capital proved unfeasible. Brazil is a big, heterogeneous country and the population may differ in some aspects between and within major states. Since all the cases were from state capitals and the controls came from different states from the same macro region, they are likely differ in some aspects that were not possible to consider in this study.

### Conclusion

Our findings highlight the need to invest in harm reduction measures such as needle exchange programs where pockets of injection drug users remain active and distribution of condoms and lubricants, which can act as barriers to the spread of HCV and other infections classically defined as sexually transmissible. HCV infection is mostly asymptomatic, and when the individual does present symptoms the infection is usually established and is associated with serious health harms such as cirrhosis and/or liver cancer. To avoid the severe forms and deaths resulting from HCV infection requires not only preventive measures but also widely available and speedy diagnosis, prompt referral to health services, and guaranteed adequate treatment.

The high efficiency of the new generation of antivirals (directly acting antivirals [DAAD]) make them essential for both the proper treatment (and eventual cure) of individual patients, as well as to decrease average viral load/infectivity at the population level, as highlighted by the abovementioned strategy endorsed by the WHO, to eliminate HCV as of 2030.

## Methods

### Study Design

The current study carried out a cross-sectional analysis of a subset of the original database from the National Crack Cocaine Survey^26^, a nationwide survey coordinated by the Oswaldo Cruz Foundation (FIOCRUZ) of the Brazilian Ministry of Health.

The National Crack Cocaine Survey (PNC, *Pesquisa Nacional sobre o Uso de Crack*, in Portuguese) aimed to describe the profile of crack users (and users of similar coca-derived substances such as freebase, hereinafter included under the term “crack users”) in Brazil’s 26 state capitals, Federal District, 9 metropolitan areas, and an additional “Brazil” stratum consisting of medium-sized and small municipalities. Time-location sampling (TLS)^43^ was the chosen methodology, which has been applied to studies of hard-to-reach populations^26,44^. After conducting a comprehensive mapping of crack use scenes in the sampled municipalities, scenes, days, and shifts were selected for visitation, using inverse sampling in the final selection stage^45^.

The eligibility criteria was: to be a Brazilian citizen, aged 18 years or older, who had used crack and/or similar substances for at least 25 days in the six months prior to the survey, which corresponds to approximately at least once a week, according to the CODAR criterion^46^ in open drug use scenes^26^. The exclusion criteria comprised individuals with severe mental health disorder (include those who were acutely intoxicated at the moment they would be interviewed), conditions that may preclude potential interviewees to understand the nature and purpose of the study and to comprehend the informed consent form.

Selected crack use scenes were visited by trained staff, including recruiters and interviewers, mostly health professionals. Individuals were approached to eligibility verification by recruiters. Those that met the eligibility criteria and signed the free and informed consent form proceeded to answer a questionnaire in a face-to-face interview. Interviewers used a pre-tested paper-and-pencil questionnaire. Data were collected in 2011 and 2012, and 7,381 persons were interviewed. Additional information about the PNC can be found in the Supplementary Material.

### Diagnostic testing

After answering the questionnaire, subjects were invited to do an HIV test (Rapid Check HIV [screening] and Biomanguinhos [confirmatory]) and an HCV test (WAMA Imuno-Rápido HCV-Kit®). Testing was accompanied by the necessary pre and post-counseling and referral for treatment in case of detection of infection/disease. Tests and counseling were performed by health professional in accordance to the Brazilian Ministry of Health norms.

HCV testing used the rapid immune test produced by Wama Diagnóstica®. Blood samples for the test were drawn by fingerstick, according to manufacturer’s instructions. Use of the rapid test aimed to simplify the field logistics, streamline the diagnosis and referral to health services, and minimize the biological risks of these procedures (venous puncture in open crack use settings has been associated with operational difficulties and biosafety risks). Under laboratory conditions, the test shows high sensitivity and specificity and adequate predictive value, as informed by the company that produces the kit and confirmed by the Brazilian health regulatory agency (ANVISA).

### Selection of study participants

The prevalence of HCV infection in crack users in open scenes in Brazil, according to the PNC survey, was 2.63% (95% CI: 1.69–4.07)^26^.

Considering this prevalence, higher than in the general population but still relatively low from the statistical point of view of a large sample, the decision was made to use a case-control study design. Cases were all the 129 participants with positive results in the rapid test for hepatitis C virus (HCV-POSITIVE), all living in state capitals. For each case, four individuals with negative HCV test results (HCV-) were randomly selected as controls, matched by sex, age category (18–30; 31–45, 46+ years), and major geographic region of the country (matching by state capital proved unfeasible). Matching by age category aimed to minimize the effect of age cohort or birth cohort, since mandatory HCV screening tests in blood banks were implemented systematically in Brazil in the 1990s^47^; the matching also attempted to provide a broad balance in exposure time, since exposure to HCV is often cumulative and extended over time.

### Observed outcome variable

The outcome variable was defined as a “positive” (serologically reactive) result in the HCV antibody test, which in the current study was the WAMA Imuno-Rápido HCV-Kit® diagnostic test.

### Covariates (Exposure)

The study initially considered the following potential risk factors associated with hepatitis C infection with available information from the PNC questionnaire: sociodemographic data, including race/color (“white”, “black”, “brown’, “indigenous/Asian-descendent”); schooling (“none”, “primary”, “secondary”, “university”); type of housing in the 30 days prior to the interview (“some type of fixed housing”, “temporary housing”, “homeless”, “other”), and main source of income in the last 30 days (“odd jobs”, “regular formal employment with benefits”, “illegal activity”, “family/partner”, “sex work”, “begging”).

As for psychoactive substance use, the variable “polydrug use” was created (“use of 1 illegal drug” vs. “use of 2 or more illegal drugs”). Polydrug use was defined as individual self-report of the use of two or more illegal drugs (which included marijuana, cocaine, ecstasy, or amphetamines) in addition to crack in the 12 months prior to the study. Alcohol and tobacco were not computed in the composition of the ‘polydrug use’ variable, because they are legally sold and used in Brazil and were reported to be used on a regular basis by the vast majority (over 90%) of the study subjects^26^, to the point of losing any discriminatory value. In addition to polydrug use, we also included the length of crack use (considered here as a dichotomous variable, i.e., up to 5 years versus more than 5 years); lifetime use of injection drugs (“yes” vs. “no”); and having shared crack-use paraphernalia in the 30 days prior to the interview (“yes” vs. “no”).

The following clinical variables were selected: result of the rapid HIV test (“positive” vs. “negative”); report of genital ulcer (“yes” vs. “no”); and report of genital warts in the 30 days prior to the interview (“yes” vs. “no”).

Risk behavior factors in the 30 days prior to the interview were: condom use (“did not have sexual relations”, “failed to use a condom at least once”, “used condoms in all sexual relations”), trading sex for drugs or money (“yes” vs. “no”) and oral and gingival sores (“yes” vs. “no”). Having any tattoos and/or piercing (“yes” vs. “no”) was also selected.

As for the inherent context in the open drug use scenes, the model considered the “shift” in which the person was recruited at the scene (“morning/afternoon” vs. “night”) and day of the week (“Monday to Thursday” vs. “Friday to Sunday”).

### Statistical analysis

We calculated the absolute and relative frequencies of the categorical variables potentially associated with the HCV test result. These associations were verified with the chi-square test. Variables with statistical significance at p-value ≤25% were included in the regression models, following the suggestion made by Sander Greenland’ classic paper on optimal procedures (to be as flexible as possible in every first round) to be putatively used in the preliminary assessment of complex datasets and respective modeling strategies^48^.

The presence of explanatory factors for HCV infection at different levels (individual and contextual) informed the choice of multilevel logistic regression models^49^. The first level is the context to which the subject belongs, specifically the time and day of the week at the crack use scene where the individual was recruited. The time of day and day of the week in which the individual was recruited were included in the model as random effects, based on the hypothesis that the potential associations between the target risk factors and the result of the rapid HCV test could differ between individuals that frequent open drug use scenes during different shifts and on different days. Another hierarchical structure was defined by the case-control matching index, defined by a unique number attributed to each case and its respective four matched controls according to sex age bracket, and major geographic region. The case-control matching index was added into the model as random effects.

HCV infection is represented in this study’s context exclusively by the rapid HCV diagnostic test result, in the absence of additional confirmatory diagnostic tests. A diagnostic test’s result is influenced by the kit’s sensitivity and specificity, besides the challenges posed by their concrete application under different operational conditions, in different contexts and populations (e.g. predominance of given strains after founder effects or uneven geographic dissemination in given populations or higher variable background infection rates in different populations)^49^. There is an abundant literature on kits’ sensitivity and specificity in different contexts and populations in a broad sense, but few ones have addressed the simple but anyway challenging issues of their proper conservation, use under unfavorable conditions and by people who may lack optimal training or have their skills challenged by real-life hurdles such as less-than-optimal conditions to read kits’ results (poor lightning, places under permanent pressure imposed by lack of running water, high humidity, etc^50^.

Thus, in addition to the traditional multilevel logistic modeling, a second set of logistic regression models were implemented, incorporating the outcome’s uncertainty (further details available in the web appendix). The diagnostic test’s sensitivity and specificity were documented by Scalione *et al*.^49^ while assessing its performance in diverse contexts. The choice was made to use these values, since the context assessed was closer to that observed in our fieldwork than that on the test’s package insert. The study thus considered point estimates of 93.23% for sensitivity (95% CI:89.38–96.01) and 99.07% for specificity (95% CI:97.32–99.81)^49^.

The results of the multilevel logistic regression models with and without incorporation of sensitivity and specificity values are presented as odds ratios (OR) and respective 95% confidence intervals.

The analyses were performed with the R software package, version 3.4.2^51^. A specific link function was created for the multilevel logistic regression model uncertainty in the outcome by providing the sensitivity and specificity. The model formulation and the scripts are available in the Supplementary Material.

### Ethical approval and informed consent

The study was approved by the Institutional Review Board (IRB) of the Sergio Arouca National School of Public Health (ENSP/FIOCRUZ), case review CAAE 0073.0.031.000-11. The IRB from ENSP/FIOCRUZ is part of the network CEP-CONEP. All research was performed in accordance with international ethical standards. Informed consent was obtained from all participants.

## Electronic supplementary material

Supplementary Information

Dataset

## Data Availability

All data generated or analyzed during this study are included in this published article (and its Supplementary Information Files).
