# Supplementary material for: The risks of HCV infection among Brazilian crack cocaine users: incorporating diagnostic test uncertainty
Source: Sci Rep. 2019 Jan 24;9:443. doi: 10.1038/s41598-018-35657-0 (PMC6346030; doi:10.1038/s41598-018-35657-0)
Supplement: Supplementary file 1 — Supplementary Information [file 41598_2018_35657_MOESM1_ESM.pdf]

**The risks of HCV infection among Brazilian crack cocaine users: incorporating diagnostic test uncertainy**

**Carolina Coutinho*[1,2], Leonardo S. Bastos[3], Jurema Corrêa da Mota[1], Lidiane Toledo[1], Katia Costa[1], Neilane Bertoni[4], Francisco I. Bastos[1]**

[1]Institute of Scientific and Technological Communication and Information in Health (ICICT), FIOCRUZ, Rio de Janeiro, Brazil.
[2]Program of Epidemiology in Public Health, Sergio Arouca National School of Public Health (ENSP), FIOCRUZ.
[3]Scientific Computing Program, FIOCRUZ
[4]Division of Epidemiology, National Cancer Institute (INCa), Rio de Janeiro, Brazil.
* coutinho.carol@gmail.com

## Supplementary Information

### I) Basic Methods and Procedures used by The National Crack Cocaine Survey (PNC, *Pesquisa Nacional sobre o Uso de Crack*, in Portuguese)

Several methods have been developed and used when the goal is to learn more about the characteristics and behaviors of hard-to-reach populations. Such sampling methods provide (or attempt to provide) an approximation of probability sampling. One such method is Time-Location Sampling (TLS). Our study used the TLS method with some modifications in the selection of potential interviewees in the last selection stage (as described in De Boni et al., 2012).

The survey was conducted in 37 geographic strata, as follows: the 26 state capitals plus the Federal District, nine metropolitan areas (Belém, Fortaleza, Recife, Salvador, Belo Horizonte, Rio de Janeiro, São Paulo, Curitiba, and Porto Alegre), and the set of the country's other municipalities, referred to here as the "Rest-of-Brazil Stratum" (ERB). The municipalities in the ERB were selected considering a matrix of neighboring municipalities and seeking to group them heterogeneously in relation to the 3-years mortality rate from external causes, obtained from Brazil's Mortality Information System (SIM) for the most

recent available period (2007-2009) at the time of the sample selection. This choice was due to the absence of a comprehensive listing of drug users and the well-documented ecological association between drug traffic and violence (in such case, homicides) in Brazil (Beato Filho et al., 2001) and elsewhere.

TLS can defined as a physical and temporal cluster sampling method (Stueve et al., 2001), generally with two or three selection stages. The National Survey on Crack Cocaine had a formative phase with the aim to provide the necessary feedback for a comprehensive sampling plan, commencing with the macro dimension, but zooming at crack cocaine scenes. To elaborate this list/mapping, in each of the selected municipalities, the study collected and triangulated information from governmental agencies (social assistance departments, law enforcement agencies, etc.) and nongovernmental organizations (NGOs) that work with this population. Our team also drew on key informants, including drug users, to identify the places where crack cocaine users gathered to consume the substance, which could also be locations for its purchase, since such activities frequently overlap.

Ideally, this exhaustive mapping of scenes and locations should profit from the concerted effort of ethnographers in different contexts, working for long periods (usually years). The literature includes drug scene ethnographies that have become classics, like the study by P. Bourgois (Bourgois, 2003).

Ethnographies, as a rule, are local (see Page & Singer, 2010). In our study, the national scope, the need to provide feedback to a TLS-based survey in a period of a few months (rather than years) meant that the work should be better described as a rapid assessment. There are numerous manuals for performing such assessments and dozens of articles summarizing their results. Although the results obviously fall short of what could be obtained from a detailed ethnography in each location, they are quite useful in the effort to map the respective sites.

Additional information was recorded at these scenes including days of the week and times when the scenes operated. This set of information is described in TLS as the venue-day-time method (VDT) (Muhib et al., 2001). This temporal information is crucially important, since the population in such scenes is highly dynamic, potentially varying in its

contingent and composition as a function of the day of the week and shift (morning/afternoon/night).

After mapping the crack cocaine scenes, we randomly selected the locations, days of the week, and shifts to be visited by the fieldwork teams, using the modified TLS method (De Boni et al., 2014). This venue-time distribution aimed to assess and register the scenes' dynamics. The goal was thus to obtain a representative sample (or rather a sample as complete as possible under real conditions on the ground) of crack cocaine users who consumed the drug in the mapped public scenes.

Some scenes proved to be totally inaccessible, for example, in favelas or other areas controlled by the drug traffic, even with the help of community health workers or facilitators for the field team.

The fieldwork team consisted of observers/recruiters, interviewers, and technicians in charge of perform rapid tests, besides supervisors in each of the capitals or designated survey areas.

The observers/recruiters, identified with ID badges and t-shirts with the FIOCRUZ logotype, received pre-generated Collection Spreadsheets, informing the location of the scene, day of the week, and the planned time (or shift) for initiating recruitment of participants.

Besides the spreadsheets, qualitative and quantitative information about the drug scenes were keyed into a Field notebook. This information included the scene's characteristics, their surrounding areas, a head count of the persons present, and the presence or absence of children (who were just counted but not interviewed due to ethical cocerns) consuming drugs at the time of the visit.

Visits were always conducted in pairs for safety reasons, but the Field notebooks were completed individually to enrich the qualitative part of the survey with different views of the same scene. These visits also aimed to verify whether the mapped location was actually a crack cocaine scene. Our team amassed over 12,000 PDF files from the Field Notebooks,

which constitute a precious source of information on the daily dynamics of crack cocaine scenes.

After initial observation of the scene, the recruitment of individual subjects began. In order to ensure random selection of users, it was stipulated that the survey should only approach individuals that were exiting a given scene (while the team remained at the scene, referring to a given venue-day-time), adopting an inverse sampling procedure (De Boni et al., 2014).

The persons that were approached answered a simplified eligibility questionnaire, and when eligible they received an invitation containing information on the venue where the interview would take place to complete the questionnaires and undergo rapid HIV and HCV tests, in addition to taking a sputum smear for TB microscopy.

The questions on eligibility included the queries as follows: i) Have you already been invited to participate in this study? ii) Are you 18 years or older? iii) Have you used crack cocaine and/or related substances for at least 25 days in the last 6 months (after PAHO CODAR criteria; available at https://www.paho.org/hq/index.php?option=com_content&view=article&id=853:2009-encuestas-comportamiento-consumidores-drogas-alto-riesgo-codar&Itemid=820&lang=en).

The eligibility questionnaire also include questions that were worded to verify whether the person recruited was the same that later came to the interview venue.

Recruited crack cocaine users were usually transported by the fieldwork team to the service that operated as the base for conducting the survey (in cars rented by the survey or belonging to institutional partners), but users also had the option of going on their own, bringing the invitation received from the fieldwork team. In the place where the interviews themselves were held, the eligibility questionnaire was applied again, since invitations given to recruited individuals but not taken with them could have been passed on to others, potentially ending up in the hands of ineligible persons. When eligible, the participant signed the free and informed consent form and proceeded to the other stages of the survey (full flowchart with respective figures available under request).

The survey's individual questionnaire was initially based in the Canadian standard assessment form, then used in a preliminary study, sponsored by the BMoH, comprising two Brazilian cities located in different macro-regions (Rio de Janeiro, Southeast and Salvador, Bahia, Northeast). Although the questionnaire as a whole was not formally validated either in Canada or Brazil, it was basically composed by standard questions used for decades in the context of Brazilian Censuses (available at: https://memoria.ibge.gov.br/sinteses-historicas/historicos-dos-censos/censos-demograficos.html) plus validated scales, such as the scale for the self-assessment of health status (Nery Guimaraes et al., 2012).

Several publications eventuated from both the Canadian assessment, nested in a multicenter cohort study (Fischer et al., 2006) and the preliminary Brazilian study (Santos Cruz et al., 2013a; Santos Cruz et al., 2013b; Cruz et al., 2013; Bertoni et al., 2014). The latter helped to improve phrasing and the overall sequence of the questionnaire, after discussion of its contents by a group of experts as well as role-playing sessions involving community leaders and the FIOCRUZ core team.

The questionnaire comprised 8 eight sections: a) social and demographic information; b) substance use; c) mobility (neighborhoods/municipalities where the person used crack cocaine); d) risk of infectious diseases associated with use (obtaining and using crack cocaine and sharing paraphernalia for use); e) sexual behavior; f) self-rated health (physical and mental); g) social and healthcare services (services used and factors that facilitated their use); and h) police and/or prison record.

Counseling was provided on testing and sexually transmitted diseases. Post-counseling was also provided when the results were returned, and individuals were referred to specialized health services when necessary.

The field team consisted of individuals with different backgrounds, depending on their role in the project. The recruiters did not require any specific training, but needed experience in or familiarity with drug issues and fieldwork. Some recruiters had experience with "harm reduction" programs/projects or were community health workers or affiliated with NGOs that worked with such issues.

Most of the interviewers had university training in psychology, social sciences, and related areas and/or had previous experience in interviewing drug users. Lab samples were always taken by health professionals properly registered with their professional boards, since they verified the results of the rapid tests.

Crack cocaine users that participated in the survey received either a meal voucher or lunchbox, according to the availability in the respective municipality.

All survey instruments were then reviewed by the field supervisor before turning them in to the project's central headquarters (located in Rio de Janeiro). The questionnaires, elaborated in Teleform® (analog-to-digital conversion software), were then scanned, reviewed, and stored in a databank in SPS[illegible] format with a subsequent verification of the data's consistency. The other data collection instruments were keyed-in using dedicated databanks (using Access®), and the Field notebooks were digitized and stored in Atlas TI®.

The sample was designed to represent the population of crack cocaine users that frequented the mapped crack cocaine use scenes and smoked the drug in pipes, cans, and other paraphernalia (not those that have uses smashed crack mixed with tobacco cigarettes or cannabis [known as "mesclado" and hard to identify as such in the absence of an in-depth, focused assessment; Gonçalves & Nappo, 2015]). Likewise, the survey did not include persons that used crack cocaine exclusively in private homes or closed institutions like prisons.

## II) The multilevel logistic regression model with uncertainty

The model consists in a logistic regression model for the rapid HCV test results where the sensitivity and specificity are added in the link function as stated by van den Hout, Heijden, and Gilchrist (2007), i.e.

$$Y_i \sim Bernoulli(\pi_i), i = 1,2, \dots, n,$$

where $Y_i$ is the HCV test result for participant $i$, and $\pi_i$ is the probability of a positive result.

We are interested in the actual probability of an HCV infection, $\theta_i$, which isdepends on the rapid HCV test's sensibility and specificity. Hence, by applying probability definitions

we have that where $\gamma_{se}$ is the rapid HCV test's sensitivity and $\gamma_{sp}$ is the rapid HCV test's specificity.

Covariates such as polydrug use, injection drug use and genital ulcers (these are the final model significant fixed effects) were added in the model together with the random effects in the following link function,

$$logit(\theta_i) = \alpha + \mathbf{x}_i^T \beta + \delta_{\text{shift}[i]} + \psi_{\text{week}[i]} + \phi_{\text{pair}[i]}$$

where $\phi_{\text{pair}}$ is the matching index random effects, and $\delta_{\text{shift}}$ and $\delta_{\text{week}}$ are, respectively the context in the open drug use scenes random effects for shift and day of week in which the person was recruited at the scene.

Scalioni et al. (2014) found the following values for rapid HCV test uncertainty for drug users: 93.23% for sensitivity (95% CI: 89.38-96.01) and 99.07% for specificity (95% CI: 97.32-99.81). These values were used in our modelling.

**R script and data**

The link function was coded in R and presented here as following R function:

```
# Link function (s = sensitivity; e = specificity)
logitse <- function(s=1, e=1)
{
  linkfun <- function(mu) qlogis((mu+e-1)/(s+e-1))
  linkinv <- function(eta) plogis(eta) * (s+e-1) + 1 - e
  mu.eta <- function(eta) (s + e - 1) *
    .Call(stats:::C_logit_mu_eta, eta, PACKAGE = "stats")
  #.Call("C_logit_mu_eta", eta, PACKAGE = "stats")
  valide ta <- function(eta) TRUE
  link <- paste("logitse(", s,",", e, ")", sep="")
  structure(list(linkfun = linkfun, linkinv = linkinv,
                 mu.eta = mu.eta, valideta = valideta, name = link),
            class = "link-glm")
}
```

This function works in glm (base), glmer (lme4) and svyglm (survey) in R 3.4.2 and 3.5.0.

```
# Multilevel model library
library(lme4)

# Reding data
load("HCVdata.RData")
```

```r
# Multilevel logistic regression
modelo.0 <- glmer( formula = HCV ~ (1|Par) + (1|semana_new) + (1|turno_ne
w) +
                      IDU + Polydrug + GenitalUlcers,
                    data = dadosHCV,
                    family = binomial())

# Multilevel logistic regression with outcome uncertainty
modelo.1 <- glmer( formula = HCV ~ (1|Par) + (1|semana_new) + (1|turno_ne
w) +
                      IDU + Polydrug + GenitalUlcers,
                    data = dadosHCV,
                    family = binomial(logitse(.9323,.9907)) )
```

Estimates for the ORs of the traditional multilevel logistic model:

```r
se.modelo.0 <- sqrt(diag(vcov(modelo.0)))
tab.modelo.0 <- cbind(Est = fixef(modelo.0),
                         LL = fixef(modelo.0) - 1.96 * se.modelo.0,
                         UL = fixef(modelo.0) + 1.96 *se.modelo.0)
(M0 <- round(exp(tab.modelo.0[-1,]),2))

##                 Est   LL    UL
## IDU            7.60 4.87 11.85
## Polydrug       1.75 1.06  2.89
## GenitalUlcers 3.91 1.29 11.89
```

Estimates for the ORs of the multilevel logistic model with uncertainty:

```r
se.modelo.1 <- sqrt(diag(vcov(modelo.1)))
tab.modelo.1 <- cbind(Est = fixef(modelo.1),
                         LL = fixef(modelo.1) - 1.96 * se.modelo.1,
                         UL = fixef(modelo.1) + 1.96 *se.modelo.1)
(M1 <- round(exp(tab.modelo.1[-1,]),2))

##                 Est   LL    UL
## IDU            8.81 5.40 14.37
## Polydrug       1.85 1.06  3.21
## GenitalUlcers 4.69 1.38 15.92
```

**References**


Beato Filho, C. C., Assunção, R. M., Silva, B. F., Marinho, F. C., Reis, I. A., Almeida, M. C. Homicide clusters and drug traffic in Belo Horizonte, Minas Gerais State, Brazil from 1995 to 1999. *Cad de Saúde Pública* **17(5),** 1163-71 (2001).

Bertoni, N., Burnett, C., Cruz, M. S., Andrade, T., Bastos, F. I., Leal, E., Fischer, B. Exploring sex differences in drug use, health and service use characteristics among young urban crack users in Brazil. *Int J Equity Health* **13(1),** 70 (2014).

Bourgois, P. I. In Search of Respect: Selling Crack cocaine in El Barrio. Cambridge, New York *Cambridge University Press* (2003).

Cruz, M., Bertoni, N., Bastos, F. I., Burnett, C., Gooch, J., Fischer, B. Comparing key characteristics of young adult crack users in and out-of-treatment in Rio de Janeiro, Brazil. *Subst Abuse Treat Prev Policy* **10**, 9-2 (2014).

De Boni, R., do Nascimento Silva, P. L., Bastos, F. I., Pechansky, F., de Vasconcellos, M. T. Reaching the hard-to-reach: a probability sampling method for assessing prevalence of driving under the influence after drinking in alcohol outlets. *PLoS One* **7(4),** e34104 (2012).

Fischer, B., Rehm, J., Patra, J., Kalousek, K., Haydon, E., Tyndall, M., El-Guebaly, N. Crack across Canada: Comparing crack users and crack non-users in a Canadian multi-city cohort of illicit opioid users. *Addiction* **101(12),** 1760-70 (2006).

Gonçalves, J. R., Nappo, S. A. Factors that lead to the use of crack cocaine in combination with marijuana in Brazil: a qualitative study. *BMC Public Health* **15,** 706 (2015).

Muhib, F. B., Lin, L. S., Stueve, A., Miller, R. L., Ford, W.L., Johnson, W. D., Smith, P. J. A venue-based method for sampling hard-to-reach populations. *Public Health Reports* **116 (Suppl 1),** 216–222 (2001).

Nery Guimarães, J. M., Chor, D., Werneck, G. L., Carvalho, M. S., Coeli, C. M., Lopes, C. S., Faerstein, E. Association between self-rated health and mortality: 10 years follow-up to the Pró-Saúde cohort study. *BMC Public Health* **12**, 676 (2012).

Page, B., Singer, M. Comprehending Drug Use: Ethnographic Research at the Social Margins. New Brunswick, N.J.: *Rutgers University Press* (2010).

Santos Cruz, M., Andrade, T., Bastos, F. I., Leal, E., Bertoni, N., Villar, L. M., Tiesmaki, M., Fischer, B. Key drug use, health and socio-economic characteristics of young crack users in two Brazilian cities. *Int J Drug Policy* **24(5)**, 432-8 (2013a).

Santos Cruz, M., Andrade, T., Bastos, F. I., Leal, E., Bertoni, N., Lipman, L., Burnett, C., Fischer, B. Patterns, determinants and barriers of health and social service utilization among young urban crack users in Brazil. *BMC Health Serv Res* **13**, 536 (2013b).

Scalioni, L. P., Cruz, H. M., De Paula, V. S., Miguel, J. C., Marques, V. A., Villela-Nogueira, C. A., Milagres, F. A., Cruz, M. S., Bastos, F., Andrade, T. M., Motta-Castro, A. R., Lewis-Ximenez, L. L., Lampe, E., Villar, L, M. Performance of rapid hepatitis C virus antibody assays among high- and low-risk populations. *J Clin Virol* **60(3),** 200-5 (2014).

Stueve, A., O'Donnell, L. N., Duran, R., San Doval, A., Blome, J. Time-space sampling in minority communities: results with young Latino men who have sex with men. *American Journal of Public Health* **91(6)**, 922–926 (2001).

Van den Hout, A., Van der Heijden, P. G. M., Gilchrist, R. 2007. The Logistic Regression Model with Response Variables Subject to Randomized Response. *Computational Statistics & Data Analysis* **51 (12),** 6060–9 (2007).
